# Patients with gynecological malignancies are similar to other IVF patients without cancer for clinical and molecular reproductive parameters and DNA damage response pattern

**DOI:** 10.1038/s41598-024-64403-y

**Published:** 2024-06-13

**Authors:** Yashar Esmaeilian, Sevgi Yusufoglu, Ece İltumur, Deniz Ugurlu Cimen, Dogan Vatansever, Cagatay Taskiran, Volkan Turan, Kayhan Yakin, Said İncir, Bulent Urman, Ozgur Oktem

**Affiliations:** 1 Research Center for Translational Medicine, Koç University, Istanbul, 34450 Turkey; 2 The Graduate School of Health Sciences, Koç University, Istanbul, 34450 Turkey; 3 Department of Obstetrics and Gynecology, School of Medicine, Koc University, Davutpasa, Topkapi, 34010 Istanbul, Turkey; 4 Department of Obstetrics and Gynecology, İstanbul Health and Technology University Faculty of Medicine, Istanbul, Turkey; 5 Department of Biochemistry, Koç University School of Medicine, Istanbul, Turkey

**Keywords:** Cancer, Cell biology, Endocrinology, Medical research, Oncology

## Abstract

This study intended to investigate if gynecological cancers compromise ovarian function and reduce the success of assisted reproduction techniques (ART). No clinical and molecular data together is available on this issue for gynecological or other organ cancers. Steroidogenic pathways and DNA damage response characteristics of the granulosa cells retrieved from the 39 gynecological cancer patients were analyzed together with their clinical ART characteristics in comparison to 31 control ART patients. Patients with gynecological malignancies were similar to the control IVF patients for the number of mature oocytes retrieved, fertilization rates and embryo development competency. Molecular analyses of the granulosa cells retrieved from these cancer patients did not detect any perturbations in gonadotropin receptor expression and response, sex steroid production, cholesterol utilization/storage and, DNA damage response pattern in comparison to control IVF patients without cancer. This study provides the first reassuring clinical and molecular combined data set that the presence of gynecological malignancy does not appear to have any detrimental effect on clinical IVF cycle characteristics and ovarian functioning at molecular level.

## Introduction

Modern combined cytotoxic chemotherapy and radiation regimens for cancer treatment have toxic effects on ovarian follicles, affecting both dormant primordials and growing follicle fractions. The cardinal pathophysiologic event of ovarian damage after cancer treatment is the apoptotic death of oocytes, granulosa cells and surrounding stroma, leading to follicle atresia and early exhaustion of the follicle stockpile^1–5^. Assisted reproduction technologies [Controlled ovarian stimulation (COS) with gonadotropin hormones for oocyte/embryo freezing] before exposure to gonadotoxic treatment modalities and/or cancer surgery is currently the most established method with the highest success rate in preserving fertility of adult women with cancer according to the most recent fertility preservation guidelines^3^. Besides the detrimental effects of chemotherapy and radiation, cancer itself may adversely affect ovarian reserve and functioning. Several studies and meta-analyses demonstrated that patients diagnosed with cancer, particularly those with hematological malignancies and those who carry germline BRCA mutations are more likely to have diminished ovarian reserve and shortened reproductive life span^6,7^. Nevertheless, available evidence regarding the impact of cancer diagnosis itself on reproductive performance is rather inconsistent largely because of heterogeneity in patient population and types of cancer^8–12^. A recent meta-analysis of 41 studies demonstrated that women with cancer may be less likely to achieve pregnancy and live birth after embryo transfer. However, there was a substantial among-study heterogeneity in the clinical indicators of the response of the ovaries to ART treatment such as duration of therapy, gonadotropin dose, cycle cancellation, total oocytes and mature oocytes retrieved^12^. Further, none of those studies evaluated ovarian functions at molecular level in cancer patients. Therefore, it is unknown if cancer itself has any detrimental effect on ovarian functioning and reduce ART success. In order to address this question and minimize the heterogeneity and confounder effects we studied only the patients with gynecological malignancies undergoing ART for oocyte/embryo freezing before cancer treatment. Clinical ART characteristics (ovarian response to stimulation, mature oocyte yield and embryo development competency) of the patients diagnosed with gynecological malignancies undergoing ovarian stimulation and the certain molecular features of the granulosa cells (GCs) obtained from these patients during oocyte retrieval were compared with standard IVF patients without cancer. For molecular analyses, retrieved granulosa cells were cultured and steroidogenic pathways and their DNA damage response characteristics were analyzed.

## Patients and methods

This study was approved by the institutional review board of Koç University (IRB# 2017.141. IRB2. 069 and IRB#2019.299.IRB2.092). All methods were performed in accordance with the relevant guidelines.

### Patients

Thirty-nine patients who were diagnosed with different gynecological malignancies and were seeking fertility preservation before exposure to gonadotoxic chemotherapy/radiation regimens and/or cancer surgery between the years 2017–2023 gave informed consent to be enrolled in the study. Of these, 25 patients were diagnosed with epithelial and non-epithelial ovarian cancers, 14 with corpus uteri and 3 with cervical malignancies (Supplemental Table 1). Healthy 31 standard IVF patients who underwent ovarian stimulation conventionally at early follicular phase of the cycle for different infertility etiologies served as controls. Ovarian stimulation was started at early follicular phase in 12, while it was started at late follicular in 13 and luteal phase in 14 cancer patients. Control IVF patients consisted of normal responding females with male factor infertility. All patients were stimulated with GnRH antagonist protocol using GnRH antagonist (Cetrorelix acetate, EMD-Serono, Istanbul, Turkiye) and human menopausal gonadotropins (Merional IBSA) or recombinant FSH (Gonal-F, Merck-Serono, Istanbul, Turkiye) and triggered with recombinant hCG (Ovitrelle, Merck-Serono, Istanbul, Turkiye). The patients with endometrial cancer underwent ovarian stimulation with aromatase inhibitor letrozole and final oocyte maturation was induced with GnRH analog (Triptorelin (Decapeptyl), Ferring, Turkiye). Oocyte retrieval was performed 36 h after triggering ovulation. Retrieved mature oocytes were either inseminated with sperm via ICSI or vitrified on the day of retrieval. Recovered luteal GCs were recovered, processed, and analyzed separately for each individual patient.

### Conventional and random start ovarian stimulation protocols

Conventional and random start ovarian stimulation protocols including those with aromatase inhibitor cycles and all other IVF procedures were performed as we described previously^13^ and provided in supplemental data.

### Isolation and culture of human luteinized granulosa cells

Luteinized granulosa cells were obtained from follicular aspirates during oocyte retrieval process and subsequently cultured separately for each patient, following the methodology we have previously described^13–15^. In brief, recovered cells were cultured for 24 h in 6-well format culture plates at a density of 100,000 cells per well using DMEM-F12 culture medium supplemented with 10% fetal bovine serum at 37 °C and 5% CO_2_.

### Viability assay

A live/dead cell assay was conducted using YO-PRO-1 (1 mM), a green-fluorescent dye that selectively stains apoptotic cells, while live cells remain unaffected. Hoechst 33342 (1 μg/mL) was used as a counterstain. The cells were imaged using a fluorescence microscope (IX71; Olympus, Tokyo, Japan) with appropriate channels to visualize the live and dead cells.

### Gene expression analysis

The RNA isolation was carried out using the Quick-RNA MicroPrep Kit (Zymo Research, USA) following the manufacturer's instructions. The RNA was then quantified by measuring the absorbance at 260 nm using the Nanodrop 2000 spectrophotometer (Thermo Fisher Scientific.) A total of 500 ng of cDNA was prepared using M-MLV Reverse Transcriptase (Invitrogen). The quantitative real-time expression of the target mRNA was detected and compared using the Light Cycler 480 SYBR Green I Master (Roche, Germany). The primers for the genes used in the study can be found in Supplemental Table 2. The means and standard deviations (SDs) were calculated from three different measurements taken for each target gene in the qRT-PCR assay. The ΔΔCt method was employed for the relative quantitation of the target genes^16–19^.

### Immunoblotting

The cells were collected, and the amount of protein was determined using the BCA protein assay kit from Thermo Fisher (Thermo Fisher Scientific Inc., USA). The protein samples were then loaded onto Mini-PROTEAN TGX gels from Bio-Rad at a concentration of 20 µg per well. The proteins were transferred onto a PVDF membrane. To prevent non-specific binding, the membrane was blocked and then incubated overnight at 4 °C with the recommended concentrations of primary antibodies. After washing the membrane, HRP-linked secondary antibodies were added and incubated for 60 min at room temperature. The specific primary and secondary antibodies used are listed in Supplemental Table 2. Finally, the blots were visualized using the Chemi-Doc MP Imaging System from Bio-Rad after washing and incubating with ECL from Thermo Fisher.

### Conventional and confocal laser immunofluorescence imaging

The cells were cultured on glass coverslips and fixed with 4% paraformaldehyde (PFA). The coverslips were washed with DPBS-Tween and then rinsed with 60% isopropanol. They were stained with Oil Red O (Sigma, USA). After rinsing with 60% isopropanol and tap water, the cells were prepared for immunofluorescence staining. Permeabilization was done using Triton X-100, and non-specific epitopes were blocked using Super Block medium (ScyTek, USA). The cells were then incubated with primary antibodies overnight at 4 °C, followed by secondary antibodies for 1 h at room temperature. The coverslips were mounted with Fluorshield mounting medium containing DAPI (Abcam, UK), and images were captured using a confocal microscope (Leica, DMI8). The specific antibodies and fluorescent dyes used in this study are listed in Supplemental Table 3. Quantitative analysis of image intensity and colocalization was performed using the ImageJ software from the National Institutes of Health (version 2.1.0/1.53). The intensity and colocalization measurements using the ImageJ software were analyzed according to the principles outlined below: Intensity measurement: intensity of selected area/vastness of selected area = mean gray value or average intensity. Colocalization measurement: colocalized particle number/nucleus number = colocalization.

### NBD-cholesterol uptake assay

The cells were stained with 1 μg/mL NBD-cholesterol and 100 nM LysoTracker (to detect lysosomes) for 1 h at 37 °C and 5% CO_2_. After washing, the cells were fixed with 4% PFA and then coated with a Fluoroshield mounting medium containing DAPI. Finally, the cells were visualized using a confocal microscope (Leica, DMI8).

### DNA damage response analysis

Human luteinized granulosa cells recovered from follicular aspirates of the control and cancer IVF patients were cultured for 24 h in 6-well format culture plates at a density of 100,000 cells per well using DMEM-F12 culture medium supplemented with 10% fetal bovine serum at 37 °C and 5% CO_2_. After 24 h. culture period the cells were treated with cisplatin (Eli Lilly and Company, USA) (40 μg/mL) for overnight and then harvested for immunoblot analysis for DNA damage response elements as we described previously^16,17^.

### Hormone assays

Estradiol (E_2_) and progesterone (P_4_) levels in culture media were determined by using electrochemiluminescence immunoassay “ECLIA” (Elecsys and Cobas immunoassay analyzers, Roche Diagnostics, USA). Lower detection limits of E2 and P were 5.00 pg/mL (18.4 pmol/mL) and 0.030 ng/mL (0.095 nmol/mL), respectively.

### Statistical analysis

Baseline demographic and IVF parameters, transcript levels of the steroidogenic enzymes, FSH/LH receptors, hormone levels and the signal intensities in immunoblotting and confocal imaging were continuous variables and therefore, expressed as the mean ± SD. ANOVA/ Bonferroni or Kruskal Wallis/Dunn post hoc tests were applied to compare the groups if data are parametric or non-parametric respectively. The sample size required for statistical significance and proper interpretation of the results was calculated based on the qRT-PCR assays and immunoblot assays. We have used the ΔΔCt method for relative quantitation of target gene mRNAs^14,15,18,19^. The mean and SD were calculated from three different readouts taken for each target gene in the RT-PCR assay. As an example, the mean and SD of the target gene StAR were calculated after three different readouts taken for each individual sample of 20–22 IVF patients, giving a total of 66 (22 × 3) readouts with SD ranging from 0.05–0.1. Similar SDs were obtained in the readouts of other target genes. Therefore, the experiment will have an 80% power to detect a difference between the means of 0.09 with a significance level of 0.05 if we had at least 20 samples. The significance level was set at 5% (P < 0.05) and the Graphpad Prism version 9 was used to analyze the data and create the figures.

## Results

The type of gynecological malignancies of the patients enrolled in the study are given in the Supplemental Table 1. Ovarian stimulation for oocyte/embryo freezing was randomly started either at the late follicular phase (n = 13) or the luteal phase (n = 14) in 27 patients with gynecological cancers. Conventional start was initiated at early follicular phase in the remaining 12 cancer patients and 31 control IVF patients.

### Comparison of the clinical IVF characteristics of the patients with gynecological malignancies vs. control patients

In this part of data analysis, all gynecological malignancies were collectively analyzed and compared with control IVF patients for the clinical IVF cycle characteristics. There were no significant differences between the groups in terms of patients’ age and serum AMH levels and daily gonadotropin dose started. However, BMI values were significantly higher in the cancer patients (Table 1). Peak serum estradiol (E_2_) levels achieved on the hCG day were significantly lower in the cancer patients compared to the controls (1456.3 ± 561.0 pg/mL vs. 1942.7 ± 709.5 pg/mL, p = 0.002). However, the other indicators of ovarian response to gonadotropin stimulation such as gonadotropin use, duration of stimulation and the number of oocytes retrieved in the cancer patients were not different from those of control IVF patients. Furthermore, the fertilization rates and embryo development competency of the oocytes retrieved from the cancer patients as assessed by day-5 blastulation rate were also comparable to those of control IVF patients (Table 1).

**Table 1:** Baseline demographic and IVF characteristics and outcomes of the patients undergoing ovarian stimulation cycle for fertility preservation. One IVF cycle for each patient.

	Control IVF patients	IVF patients with gynecological malignancies	p
n	31	39	
Female age (y)	34.0 ± 3.7 (29–39)	35.2 ± 2.9 (29–39)	0.164
BMI (kg/m^2^)	21.7 ± 2.1 (18.3–26.0)	23.5 ± 1.8 (20.3–27.5)	0.001
AMH	1.9 ± 0.6 (0.9–3.3)	1.9 ± 0.7 (0.7–3.2)	0.864
Duration of stimulation	10.3 ± 1.1 (8–12)	10.8 ± 2.1 (7–16)	0.198
Daily dose of GN (IU)	343.5 ± 88.3 (225–450)	352.0 ± 90.5 (225–450)	0.796
Peak E_2_ (pg/mL)	1942.7 ± 709.5 (560–3148)	1456.3 ± 561.0 (587–2996)	0.002
Total number of oocytes	12.2 ± 5.5 (4–22)	10.0 ± 4.6 (3–20)	0.077
MII oocytes	9.3 ± 4.9 (3–19)	8.0 ± 3.9 (3–17)	0.229
MII oocyte/total oocyte ratio (%)	75.4 ± 16.4 (50–100)	81.2 ± 14.0 (50–100)	0.127
Fertilization rate after ICSI (%)*	81.3 ± 8.3 (69.2–94.7)	82.0 ± 17.4 (37.5–100)	0.906
Day 5 blastulation rate (%)*	45.7 ± 19.4 (13.3–83.3)	47.1 ± 12.9 (23.1–66.7)	0.833

IVF characteristics and outcomes of the patients undergoing ovarian stimulation cycle for fertility preservation. One IVF cycle for each patient.

Data are mean ± SD (range).

p values by *t*-test.

*14 women in the control group, 12 women in the gynecological cancer group had embryo cryopreservation.

*BMI* body mass index, *AMH* anti-Mullerian hormone, *GN* gonadotropin, *E*_*2*_ estradiol, *MII* metaphase-II.

### The impact of ovarian and other gynecological malignancies on IVF outcomes

All gynecological malignancies were first divided into two groups as ovarian (n = 25) and other gynecological malignancies (n = 12), and then a subgroup analysis was conducted to investigate if there is an impact of ovarian malignancy on IVF characteristics (Table 2). We observed that patients with malignant ovarian tumors had significantly lower peak E_2_ levels on the hCG day compared to control IVF patients. However, total and daily gonadotropin dose, the total and mature oocyte number, and fertilization and embryo development competency in these patients were not different from control IVF patients and those with other gynecological malignancies, suggesting that the presence of ovarian malignancy did not appear to have any detrimental effect on ovarian response to gonadotropin stimulation (Table 2).

**Table 2:** Subgroup analysis of the patients with malignant ovarian tumors vs. other gynecological cancers.

	Control group	Primary ovarian malignancies	Other gynecological malignancies	P
n	31	25	14	
Female age (y)	34.0 ± 3.7 (27–39)	35.5 ± 2.7 (30–39)	33.9 ± 2.8 (29–39)	0.216
BMI (kg/m^2^)	21.7 ± 2.1 (18.3–26.0)^a^	23.7 ± 1.8 (20.3–27.5)^a^	23.1 ± 1.8 (20.8–26.7)	0.001
AMH	1.9 ± 0.6 (0.9–3.3)	2.0 ± 0.7 (0.9–3.2)^b^	1.5 ± 0.6 (0.7–2.9)^b^	0.043
Duration of stimulation	10.3 ± 1.1 (8–12)	10.6 ± 2.3 (7–16)	11.3 ± 2.2 (9–15)	0.213
Daily dose of GN (IU)	343.5 ± 88.3 (225–450)	353.0 ± 193.8 (225–450)	350.0 ± 66.6 (300–450)	0.965
Peak E_2_ (pg/mL)	1942.7 ± 709.5 (560–3148)^c^	1340.0 ± 489.4 (587–2502)^c^	1698.7 ± 642.2 (747–2996)	0.003
Total number of oocytes	12.2 ± 5.5 (4–22)	9.8 ± 4.9 (3–20)	10.4 ± 4.1 (6–20)	0.201
MII oocytes	9.3 ± 4.9 (3–19)	7.9 ± 4.2 (3–17)	8.3 ± 3.4 (5–16)	0.467
MII oocyte/total oocyte ratio (%)	75.4 ± 16.4 (50–100)	80.5 ± 15.0 (50–100)	82.4 ± 12.2 (60–100)	0.296
Fertilization rate after ICSI (%)*	81.3 ± 8.3 (69.2–94.7)	81.3 ± 21.5 (37.5–100)	82.9 ± 12.0 (68.7–100)	0.973
Day 5 blastulation rate (%)*	45.7 ± 19.4 (13.3–83.3)	50.2 ± 16.2 (23.1–66.7)	42.7 ± 5.1 (36.4–50)	0.741

Data are mean ± SD (range).

p values by ANOVA test with Bonferroni post hoc comparison, ^a^p = 0.002, ^b^p = 0.038, ^c^p = 0.001.

*14 women in the control group, 7 women in the ovarian cancer group and 5 women in the study group had embryo cryopreservation.

*BMI* body mass index, *AMH* anti-Mullerian hormone, *GN* gonadotropin, *E*_*2*_ estradiol, *MII* metaphase-II.

### Comparison of the conventional vs. random start protocols for IVF characteristics

In 27 patients with gynecological cancers, ovarian stimulation for oocyte/embryo freezing was started either at the late follicular phase (n = 13) or the luteal phase (n = 14) while it was started at early follicular phase in the remaining 12 cancer patients and 31 control IVF patients. Ovarian response characteristics (the number of oocytes retrieved and embryo developmental competency), and fertilization and blastulation rates of the cancer patients undergoing random start protocol did not show any meaningful differences from their counterparts and control IVF patients stimulated conventionally at early follicular phase (Supplemental Table 4).

### Molecular analysis-1: viability and steroidogenic function assays

**Figure 1:**
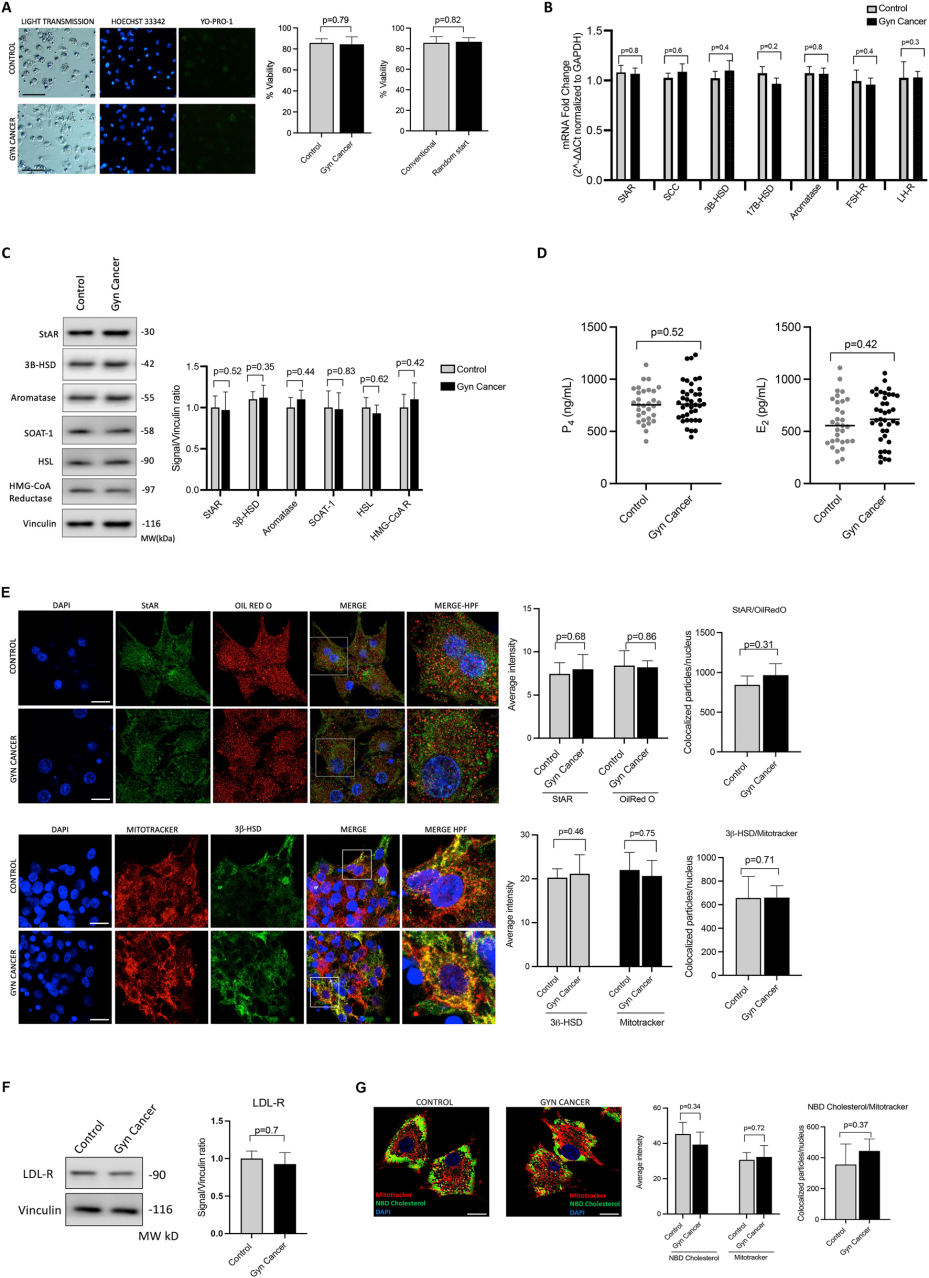
Viability, steroidogenic function, and gonadotropin receptor expression status analyses in the granulosa cells of the control and cancer IVF patients. Cell viability assay with Yo-PRO-1 staining (**A**) and the percentage of viable cells are depicted as graphic bars (to the right of the image). Comparison of the transcripts of the steroidogenic enzymes and gonadotropin receptors with qRT-PCR assay (**B**). Protein expression analysis of the steroidogenic enzymes with immunoblotting (**C**) and quantitative comparison of the signal intensities shown in the graphic bar (to the right of the image). Comparison of the in-vitro E_2_ and P_4_ production of the cell (**D**). Confocal image analyses of the cells after double-staining for cholesterol-carrying protein StAR (green signal) and Oil Red O (intracellular lipid marker, red signal) and their co-localization in low and high magnification merge images (upper panel); and for the steroidogenic enzyme 3β-HSD (green signal) and mitochondria (mitotracker: red signal) and their co-localization in low and high magnification merge images (lower panel). The average signal intensity and co-localizations are expressed as graphic bars to the right of the images (**E**). Nucleus stain is DAPI (blue signal) (DAPI). Scale bar: 20 μm. Protein expression analysis of the LDL receptor (LDL-R) of the luteinized granulosa cells in the control and cancer patients is shown by immunoblot analysis (**F**) and a quantitative comparison of the signals is shown as a graphic bar to the right of the image. Confocal image of the luteinized granulosa cells double-stained for mitochondria (mitotracker, red signal) and fluorescent cholesterol analog NBD cholesterol (green signal) (**G**). Nucleus stain is DAPI (blue signal). The average signal intensity and co-localizations are shown as graphic bars (to the right of the image). Scale bar: 10 μm. *StAR* steroidogenic acute regulatory enzyme, *SCC* side chain cleavage enzyme, *3β-HSD* 3-beta hydroxy steroid dehydrogenase, *17β-HSD* 17-beta hydroxy steroid dehydrogenase, *FSH-R* follicle stimulating hormone receptor, *LH-R* luteinizing hormone receptor, *LDL* low-density lipoprotein, *HMG-Co-A* *reductase* 3-hydroxy 3-methylglutaryl Co-A reductase, *HSL* hormone sensitive lipase, *SOAT-1* sterol O-acyltransferase 1.

The viability index analysis of the luteinized granulosa cells (GCs) that calculates the viable and apoptotic using intravital fluorescent carbocyanine dye Yo-PRO-1 fractions demonstrated that the cancer patients and standard IVF patients have similar viability index regardless of the presence of gynecological malignancy and stimulation protocol (conventional vs. random start) (Fig. 1A).

Next, we compared the transcript levels of the steroidogenic enzymes and gonadotropin hormone receptors in the GCs using qRT-PCR method and found no significant differences between the cancer and control IVF patients for the expression of the steroidogenic enzymes StAR, SCC, 3β-HSD, 17β-HSD, aromatase, and FSH and LH receptors (Fig. 1B). Quantitative immunoblot analysis for the expression of the steroidogenic enzymes (StAR, 3β-HSD, and aromatase) and other enzymes that are responsible for de novo cholesterol synthesis [(HMG-Co-A reductase (3-hydroxy 3-methylglutaryl Co-A reductase)], utilization (hormone sensitive lipase, HSL) and storage [ACAT-1 (Acetyl-Coenzyme A acetyltransferase 1; also known as SOAT-1 (Sterol O-acyltransferase 1)] demonstrated similar expression patterns in the control and cancer patients (Fig. 1C). Consistent with these findings, a comparison of the in-vitro steroidogenic function of the GCs did not show any meaningful differences between the control vs. cancer patients for E_2_ and P_4_ productions after 24 h culture period (Fig. 1D).

In another set of experiments, we analyzed the expression/staining and co-localization patterns of the steroidogenic enzymes (StAR and 3β-HSD) with mitochondria and intracellular lipids (Oil Red staining) using confocal immunofluorescence microscopy and found no difference between the control vs. cancer patients (Fig. 1E). In immunoblot analysis, the expression of LDL receptor, which is the main form of receptor for cholesterol uptake was also comparable between the control vs. cancer patients. In line with this finding NBD cholesterol (fluorescent cholesterol analog) uptake and its co-localization with mitochondria was similar between the groups (Fig. 1F,G).

### Molecular analysis-II: comparison of DNA damage response patterns and apoptosis to cisplatin

**Figure 2:**
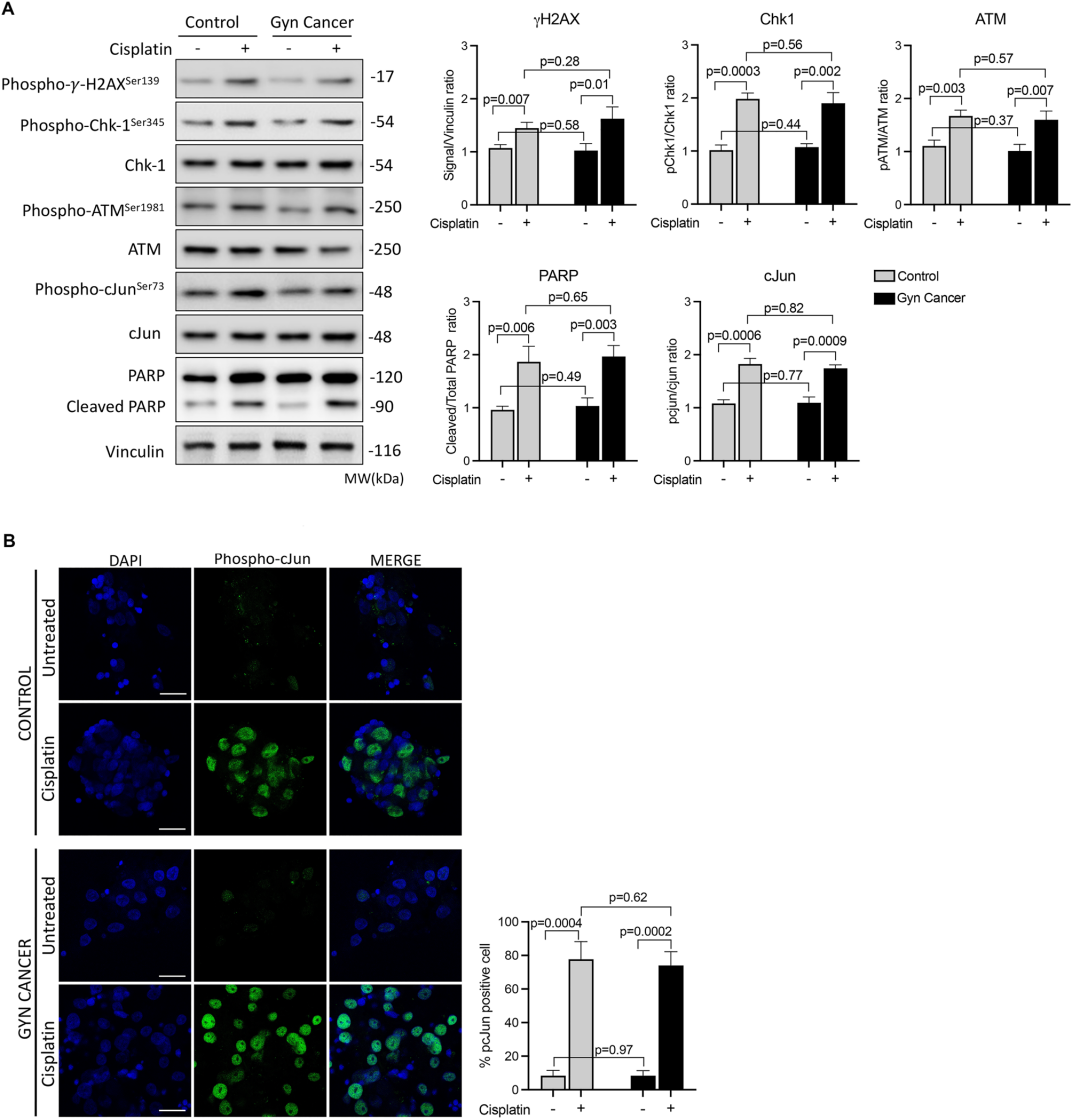
Analysis of DNA damage response patterns. Quantitative immunoblot analysis of the luteinized granulosa cells of the control and cancer patients treated with cisplatin for the comparison of the expression of the indicated DNA damage response elements. Quantitative comparison of the signal intensities is shown as the graphic bars to the right of the images (**A**). *γ-H2AX*^*Ser139*^ Gamma-H2A histone family member X, *Chk-1* Check-1 protein, *ATM* ataxia telangiectasia mutated, *PARP* poly (ADP-ribose) polymerase. Confocal image analysis of the luteinized granulosa cells stained for the phosphorylated form of c-Jun (phospho-c-Jun^Ser73^) (green signal) as evidence for SAP/JNK activation following treatment with cisplatin in the control and cancer patients. The nuclear stain is DAPI (blue signal). Scale bar: 20 μm (**B**).

In the last set of experiments, we analyzed cisplatin-induced DNA damage response (DDR) patterns and the activation of stress activated protein kinase SAPK/JNK pathway in the GCs. In the GCs of the control patients, cisplatin treatment caused genomic damage by inducing DNA double strand breaks as evidenced by up-regulated expression of the phospho-γ-H2AX^Ser139^ in immunoblotting (Fig. 2A). The drug also triggered the activation of stress activated protein kinase/JNK pathway (phosphorylated form of cjun^Ser73^) and DNA damage check point kinases (ATM^Ser1981^ and Chk-1^Ser345^) and triggered apoptosis (PARP cleavage) in immunoblot analysis. The same pattern of response to DNA damage and apoptosis were observed in the GCs of the patients with gynecological malignancies (Fig. 2A). Confocal image analysis revealed that there is an extensive activation of JNK pathway after cisplatin treatment as demonstrated by strong nuclear staining of phospho-cJun^Ser73^ in the granulosa cells of both control and cancer IVF cells (Fig. 2B).

## Discussion

To date, no study has collectively analyzed clinical IVF characteristics, molecular steroidogenic features and DNA damage response (DDR) patterns in patients with gynecological malignancies in comparison to standard IVF patients. One question still remains to be answered as to whether gynecological malignancy itself has any detrimental effect on IVF cycle characteristics and causes any subtle molecular perturbations in the steroidogenic function and DNA damage response patterns. To fill this gap, we conducted this study and demonstrated that clinical IVF characteristics of patients with gynecological malignancies undergoing ovarian stimulation are comparable to those of otherwise healthy IVF patients without cancer regardless of the stimulation protocol (conventional vs. random start). Detailed molecular analyses did not reveal any aberrations in the steroidogenic function and DNA damage response of the granulosa cells. Furthermore, in the subgroup analyses of patients with malignant ovarian tumors and other gynecological malignancies, neither clinical IVF data nor molecular features of the GCs obtained showed meaningful differences when compared to each other and standard IVF patients.

Gynecological malignancies, namely, uterine corpus, cervix and ovary are the most common genital tumors in females^20^. Although fertility-sparing surgery became a viable option at early stages of certain gynecological cancers, every woman who is diagnosed with gynecological malignancies and has not completed their childbearing should still be counseled for fertility preservation prior to cancer therapy, and the most appropriate strategy should be offered. In this context, there has been increasing use of unconventional (random start) ovarian stimulation protocols in recent years as more young women diagnosed with cancer seek fertility preservation for oocyte/embryo freezing before receiving gonadotoxic chemotherapy regimens and/or radiation or surgery^13,21^. Existing data indicate that ovarian stimulation can be initiated at any time regardless of the phase of the menstrual cycle which can effectively shorten the time to oocyte retrieval. The number of oocytes retrieved in random start cycles and their competency for euploid embryo development are also comparable to conventional stimulation protocols, proving that these protocols are a viable option for fertility preservation in female patients requiring urgent cancer treatment^13,21–28^. We have recently demonstrated that clinical IVF outcomes and molecular steroidogenic features of granulosa cells retrieved from random start cycles are not different from conventional IVF cycles started at early follicular phase in patients with different types of cancers^13^.

Platinum drugs cisplatin and carboplatin act as a DNA cross-linking agent that interferes with DNA repair mechanisms, blocks cell division and elicits DNA damage (including DNA adducts and double strand breaks)^5,17,29,30^. Although cisplatin is less toxic than alkylating agents, its moderate ovarian toxicity can still cause follicle atresia and induce vascular damage in the human ovary as shown by both in vitro studies and in vivo human ovarian xenograft models^16,17,31^. The latter model demonstrated that intraperitoneal administration of cisplatin to the xenografted nude mice resulted in a significant decline in primordial follicle count in the grafts and a decrease in serum AMH levels^17^. Cisplatin can also directly induce oocyte apoptosis in human ovary through activation of TAp63α, the oocyte-specific isoform of p63 following DNA damage^17,32,33^.

Since platinum-based combination chemotherapy protocols are the mainstay in the treatment of many gynecological malignant tumors, we selected cisplatin to analyze the DNA damage response (DDR) patterns of the GCs to this drug in the cancer patients including those with malignant ovarian tumors. We did not observe any notable difference in the DDR pattern of the granulosa cells between the control vs. gynecological cancer patients, suggesting that gynecological malignancy itself does not appear to cause any change in cisplatin-induced DNA damage response. Although these findings do not necessarily indicate that the oocytes/ovaries of the cancer patients have the same sensitivity to cisplatin as non-cancer control patients our data is still important as it for the first time provides molecular evidence for the similarity of genotoxic-stress induced DNA damage/cell cycle sensor activation and apoptosis of the granulosa cells of the gynecological cancer vs. control IVF patients.

Our data and the findings of the previous studies^13,24–26^ appear to be reassuring for female cancer patients seeking oocyte/embryo freezing to preserve their fertility. Nevertheless, caution should still be exercised when counseling patients with gynecological or other malignancies because our patient cohort consisted of small number of good prognosis early-stage cancer patients with normal ovarian reserve who had their oocytes/embryos frozen prior to chemotherapy and/or radiation. Previous exposure to gonadotoxic chemotherapy regimens or radiation and/or co-existing ovarian pathologies or infertility etiologies, previous ovarian surgery and/or any other systemic diseases might cause differences in terms of IVF parameters/outcomes and the molecular characteristics analyzed here. Limited data is available regarding the safety of ovarian stimulation for the recurrence risk of gynecological cancers^34^. The use of aromatase inhibitors during ovarian stimulation aims to reduce endogenous estrogen production in patients with estrogen sensitive gynecological tumors^35,36^. Studies with a larger number of patient cohorts preferably with a specific cancer type will provide more reliable data on the safety and effectiveness of these ovarian stimulation protocols on reproductive outcome and cancer prognosis. In-vitro nature of the study is another limitation as it might not reliably represent gonadal functioning in-vivo. Although control IVF patients underwent IVF for male factor infertility, did not have cancer and had normal ovarian function and reserve, it is unclear if any other undetected molecular aberrations related to other infertility etiologies would affect the results.

## Conclusion

Our data provides clinical and molecular evidence that gynecological cancer patients appear to have equally good prospects for fertility preservation as standard IVF patients without any demonstrable detrimental effect of the diagnosis of gynecological malignancy on the clinical IVF and molecular reproductive parameters analyzed.

### Supplementary Information

Supplementary Information.

## Data Availability

The datasets generated and/or analyzed during the current study are available from the corresponding author on reasonable request to ooktem@ku.edu.tr.
