# Supplementary material for: Patients with gynecological malignancies are similar to other IVF patients without cancer for clinical and molecular reproductive parameters and DNA damage response pattern
Source: Sci Rep. 2024 Jun 13;14:13628. doi: 10.1038/s41598-024-64403-y (PMC11176373; doi:10.1038/s41598-024-64403-y)
Supplement: Supplementary file 1 — Supplementary Information. [file 41598_2024_64403_MOESM1_ESM.docx]

**SUPPLEMENTAL INFORMATION**

**Conventional start IVF cycles**

COS was initiated on days 2-4 of the menstrual cycle in the conventional start IVF group.

The starting dose of human menopausal gonadotropins was determined according to the patient's age, body mass index (BMI), and ovarian reserve assessment by anti-Mullerian hormone (AMH) level and antral follicle count. Dosages were adjusted according to serum E_2_ levels and follicle growth kinetics during ultrasonography examination. GnRH antagonist (Cetrorelix acetate, EMD-Serono, Istanbul Turkiye) was added to prevent premature ovulation when the lead follicle measured ≥12 mm and was continued until the day of ovulation trigger. Final oocyte maturation was induced with 250 μg recombinant hCG (Ovitrelle, EMD-Serono, Istanbul, Turkiye).

**Random start IVF cycles**

The phase of menstrual cycle was determined by the onset of the last menstrual period reported by the patient, ultrasound assessment, and serum progesterone (P_4_) concentrations for the patients who did not present near the first day of menstrual bleeding and therefore was scheduled for random start COS. The late follicular phase was defined as the phase after menstrual cycle day 7 with appearance of a dominant follicle >13mm and a serum P_4_ level <1.5ng/mL. The luteal phase was determined by serum P_4_ > 3ng/mL with emergence of corpus luteum on ultrasound. Serum E_2_, P_4_, and LH levels were measured for each patient during every visit.

In the late follicular phase COS, ovarian stimulation was started without GnRH antagonist when the cohort of antral follicles trailing the dominant follicles are <12mm and remain <12mm before the LH surge. GnRH antagonist was added when antral cohorts reached >12mm to suppress secondary LH surge. If the antral cohort had already reached 12mm at the initiation of ovarian stimulation GnRH antagonist was added and continued until the day of ovulation trigger. For the patients who presented in the luteal phase, ovarian stimulation was started in the absence of GnRH antagonist, which was added later in the cycle when growing antral cohort reached >12mm and continued until the day of ovulation trigger.

**Aromatase inhibitor (Letrozole) IVF cycles**

In patients with endometrial cancer aromatase inhibitor letrozole (5mg/day per oral route) was initiated with gonadotropin stimulation in both conventional and random start IVF cycles and continued until ovulation trigger. Ovulation was induced with a GnRH analog in these patients.

**IVF laboratory procedures**

Oocyte retrieval was undertaken 36 h after the administration of hCG and performed under general anesthesia using a 17-gauge double lumen needle (Cook Ireland Ltd, Limerick, Ireland). The oocytes were collected and washed in G-MOPS Plus medium (G-MOPS Plus, Vitrolife, Sweden) and were cultured in G-IVF Plus (G-IVF Plus, Vitrolife) medium at 6.5% CO_2_, 5 % O_2_ concentration and at 37 °C for 2 hours before oocyte denudation. Oocyte cumulus complexes were denuded after at least two hours of incubation. Cumulus cell removal was carried out using hyaluronidase at a concentration of 10 IU/ml (HYASE-10X, Vitrolife) and mechanical pipetting. Following maturation assessment, all metaphase II (MII) oocytes were fertilized by ICSI. Immediately after ICSI, the injected oocytes were placed individually in pre-equilibrated culture dishes. Embryo slides (EmbryoSlide; Unisense Fertilitech A/S) were pre-equilibrated with GTL (GTL, Vitrolife) in 12-well dishes under Ovoil (Ovoil, Vitrolife) at 37 °C and 6.5 % CO_2_, 5.0 % O_2_ in a time lapse incubator (EmbryoScope). Fertilization was assessed 16-18 hours after ICSI, and the presence of two pronuclei with two polar bodies represented normal fertilization. All embryos were cultured in 37°C, 6.5 % CO_2_ and 5 % O_2_ up to the blastocyst stage. The blastocysts were evaluated according to the degree of expansion, quality of the inner cell mass and trophectoderm cells [1].

The Cryotop method (Kitazato) was used for vitrification and warming of oocytes and embryos as previously as described by Kuwayama [2].

[1] Gardner DK, Schoolcraft WB. Culture and transfer of human blastocysts. Curr Opin Obstet Gynecol. 1999;11:307-11.

[2] Kuwayama M, Vajta G, Kato O, Leibo SP. Highly efficient vitrification method for cryopreservation of human oocytes. Reprod Biomed Online. 2005;11:300-8.

**SUPPLEMENTARY TABLES**

**Supplemental Table 1:** Cancer types of the patients undergoing conventional and random start controlled ovarian stimulation cycle for fertility preservation.

| **Malignancy** | **Conventional**  **IVF cycles**  **(Early follicular phase start)** | **Random start IVF cycles** | |
| --- | --- | --- | --- |
|  |  | **Late Follicular Phase Start** | **Luteal Phase Start** |
| Uterine malignancies  (n=11)   - Endometrial   Cancer (9)   - Mixed   Mullerian tumor (1)   - Clear cell (1)   carcinoma | 5  (4)  (1)  (0) | 3  (3)  (0)  (0) | 3  (2)  (0)  (1) |
| Ovarian malignancies  (n=25)   - Serous: (8) - Mucinous: (5) - Endometrioid: (6) - Clear cell: (2) - Granulosa cell: (2) - Germ cell: (2) | 6  (2)  (1)  (2)  (0)  (0)  (1) | 9  (3)  (2)  (2)  (1)  (1)  (0) | 10  (3)  (2)  (2)  (1)  (1)  (1) |
| Cervical cancer  (n=3) | 1 | 1 | 1 |
| Total | 12 | 13 | 14 |

**Supplemental Table 2:** qRT-PCR primers were used in this study.

| **Gene** |  | **3’-Sequence-5’** |
| --- | --- | --- |
| GAPDH | Forward | ATGGAAATCCCATCACCATCTT |
|  | Reverse | CGCCCCACTTGATTTTGG |
| StAR | Forward | AAACTTACGTGGCTACTCAGCATC |
|  | Reverse | GACCTGGTTGATGATGCTCTTG |
| SCC | Forward | CAGGAGGGGTGGACACGAC |
|  | Reverse | AGGTTGCGTGCCATCTCATAC |
| 3β-HSD | Forward | GCCTTCAGACCAGAATTGAGAGA |
|  | Reverse | TCCTTCAAGTACAGTCAGCTTGGT |
| 17β-HSD | Forward | TGGGGTCCACTTGAGCCTGAT |
|  | Reverse | TGCTGTGGGCGAGGTATTGG |
| Aromatase | Forward | GGTCACCACGTTTCTCTGCT |
|  | Reverse | GCAAGCTCTCCTCATCAAACCA |
| FSH-R | Forward | TTTCAAGAACAAGGATCCATTCC |
|  | Reverse | CCTGGCCCTCAGCTTCTTAA |
| LH-R | Forward | TTGAACTGAGGTTTGTCCTCACCA |
|  | Reverse | GGCCTCAGGGTTGATGTAGAGC |

**Supplemental Table 3:** Antibodies and fluorescent dyes were used in the study.

| **Antibody** | **Manufacturer** | **Cat. No.** |
| --- | --- | --- |
| StAR | Santa Cruz Biotechnology | sc166821 |
| 3β-HSD | Santa Cruz Biotechnology | sc100466 |
| Vinculin | Santa Cruz Biotechnology | sc25336 |
| Aromatase (CYP19A) | Santa Cruz Biotechnology | sc-374176 |
| Mitotracker | Invitrogen | M22425 |
| NBD-Cholesterol | Invitrogen | N1148 |
| Hoechst 33342 | Cell Signaling | 4082 |
| SOAT-1 | Cell Signaling | 35695 |
| HSL | Abcam | ab45422 |
| HMG-CoA Reductase (HMGCR) | Abcam | ab174830 |
| LDL-R | Cayman Chemicals | 10007665 |
| ATM | Abcam | ab226985 |
| Phospho-γ HH2AX^Ser139^ | Millipore (MA, USA) | 05-636 |
| Phospho-Chk-1Ser345 | Cell Signaling | 2348 |
| Chk-1 | Cell Signaling | 2360 |
| Phospho-ATM (Ser1981) | Cell Signaling | 4526 |
| Phospho-c-Jun (Ser73) | Cell Signaling | 3270 |
| c-Jun | Cell Signaling | 9165 |
| Cleaved PARP | Cell Signaling | 5625 |
| Goat anti-Mouse Alexa Flour 594 secondary antibody | ThermoFisher | A11005 |
| Goat anti-Rabbit Alexa Flour 594 secondary antibody | ThermoFisher | A11012 |
| Goat anti-Mouse Alexa Flour 488 secondary antibody | ThermoFisher | A11001 |
| Goat anti-Rabbit Alexa Flour 488 secondary antibody | ThermoFisher | A11008 |
| Anti-Mouse IgG, HRP-linked antibody | Cell Signaling | 7076 |
| Anti-Rabbit IgG, HRP-linked antibody | Cell Signaling | 7074 |

**Supplemental Table 4:** IVF characteristics and outcomes of the gynecological oncology patients undergoing conventional or random start ovarian stimulation protocols for fertility preservation. One IVF cycle for each patient.

|  | **Conventional start** | **Random start** | **p** |
| --- | --- | --- | --- |
| n | 43 | 27 |  |
| Female age (y) | 35.5±2.9 | 35.0±3.0 | 0.610 |
| BMI (kg/m2) | 23.7±1.6 | 23.3±1.9 | 0.482 |
| AMH | 2.0±0.8 | 1.8±0.7 | 0.397 |
| Duration of stimulation | 10.1±2.1 | 11.4±2.0 | 0.064 |
| Daily dose of GN (IU) | 378.3±119 | 334.1±79.3 | 0.424 |
| Peak E_2_ (pg/mL) | 1241.8±345.9 | 1602.6±636.0 | 0.053 |
| Total number of oocytes | 8.8±4.9 | 10.1±4.2 | 0.186 |
| MII oocytes | 6.7±3.6 | 8.9±3.9 | 0.080 |
| MII oocyte/total oocyte ratio (%) | 77.8±15.4 | 83.4±12.8 | 0.236 |
| Fertilization rate after ICSI (%) | 81.5 ±25.9 | 82.3 ±10.4 | 0.940 |
| Day 5 blastulation rate (%) | 54.5 ±13.3 | 41.7 ±10.4 | 0.091 |

Data are Mean ± SD, p values by t-test

BMI: Body mass index; AMH: Anti-Mullerian hormone; GN: Gonadotropin; E_2_: Estradiol; MII: Metaphase-II
